# Diagnosis and treatment of isolated rib Langerhans cell histiocytosis in an adult: A case report

**DOI:** 10.3389/fsurg.2023.1084137

**Published:** 2023-02-22

**Authors:** Yaxin Guo, QiFeng Huang

**Affiliations:** Jinhua Hospital, Zhejiang University School of Medicine, Jinhua, China

**Keywords:** Langerhans cell histiocytosis, PET/CT, SPECT, surgery, rib

## Abstract

Langerhans cell histiocytosis (LCH) was first proposed in 1987 to define the disorder characterized by the proliferation of abnormal Langerhans cells. It is more likely to occur in children younger than 15 years of age. Single-site and single-system LCH of rib is rare in adults. We present a rare case of isolated rib LCH in a 61-year-old male patient and expound the diagnosis and treatment of the disease. A 61-year-old male patient who presented with a 15-day history of dull pain in the left chest was admitted to our hospital. PET/CT image showed obvious osteolytic bone destruction and abnormal fluorodeoxy-glucose (FDG) uptake (maximum standardized uptake value: 14.5) in the right fifth rib with local soft tissue mass formation. The patient was eventually confirmed the diagnosis of LCH by immunohistochemistry stain and treated with rib surgery. A thorough review of the literature regarding diagnosis and treatment of LCH is presented in this study.

## Introduction

Langerhans cell histiocytosis (LCH) was first proposed in 1987 to define the disorder characterized by proliferation of abnormal Langerhans cells (1). Historically, LCH included three separate syndromes: Letterer–Siwe disease, a fulminant clinical syndrome, including hepatosplenomegaly, lymphadenopathy, bone lesions, skin rash, and pancytopenia; Hand–Schuller–Christian disease, identified by the typical triad of bone lesions, exophthalmos, and polyuria; and eosinophilic granuloma, characterized by one or more bone lytic lesions (2). Depending on the organ involvement, the current clinical classification of LCH can be either a single-system or a multisystem disease (3). LCH, Erdheim–Chester disease (ECD), and extracutaneous juvenile xanthogranuloma (JXG) were proposed in a single group based on clinical and molecular relevance (4).

LCH is rare. It is more likely to occur in children younger than 15 years of age; the incidence in adults is about 1/10 of that in children (5). LCH may affect any organ, such as bone marrow, lungs, liver, spleen, lymph nodes, gastrointestinal (GI) tract, and the pituitary gland. Clinical symptoms of LCH are related to organ involvement at the time of presentation. The most common presentation is rash. Other symptoms such as bone pain, pulmonary symptoms or lymphadenopathy, hepatosplenomegaly, and diabetes insipidus can also be present when corresponding organs are involved. The organ involvement patterns of adults and children are similar while the multisystem disease of adults may evolve slowly with few symptoms (6).

The cause of LCH remains unknown; however, the suggestion that LCH is either a reactive or neoplastic process has been widely accepted. The tumor hypothesis suggests that the disease is associated with the BRAF gene mutation, and it typically responds to chemotherapy. Another hypothesis implies that LCH is a reactive disorder, which leads to abnormal reactions of Langerhans cells and T lymphocytes (7). The diagnosis of LCH relies on the biopsy of the involved site. The presence of cytoplasmic Birbeck granules on electron microscopic examination or of a clonal neoplastic proliferation with the expression of CD1a, CD207 (Langerin), and S-100 by immunohistochemistry is the golden diagnosis standard for LCH (8). For imaging examinations, they also play an important role in the evaluation of the disease. CT scan is invaluable for assessing the hypothalamic–pituitary area. Whole-body magnetic resonance imaging (WB-MRI), for its good soft tissue contrast and the absence of exposure to ionizing radiation, has been widely used in oncologic indications, including LCH. The bone scan is often additionally used to evaluate the extent of skeletal lesions. PET/CT is another option for determining the extent of LCH in the initial evaluation and follow-up, and now also being routinely used to monitor disease during treatment (9).

We present here a rare case of a 61-year-old male patient with a solitary LCH lesion in the rib evaluated by ^99m^Tc-methylene diphosphonate single photon emission computed tomography, ^99m^Tc-MDP SPECT and ^18^F-fluorodeoxy-glucose positron emission tomography/computed tomography, ^18^F-FDG PET/CT.

## Case report

**Figure 1:**
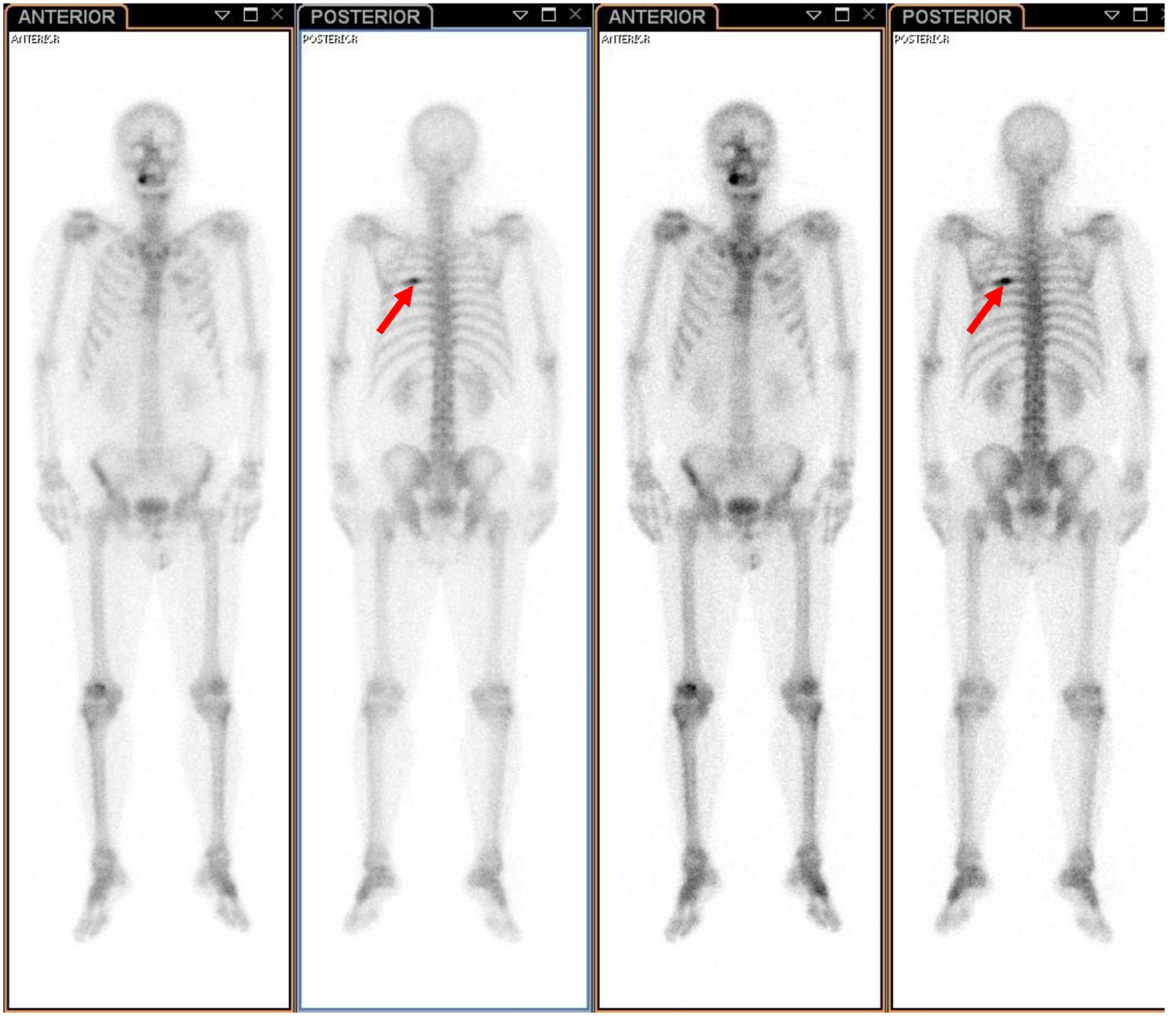
^99m^Tc-MDP SPECT revealed a lesion with high uptake of ^99m^Tc-MDP in the left fifth rib (indicated by red arrows).

**Figure 2:**
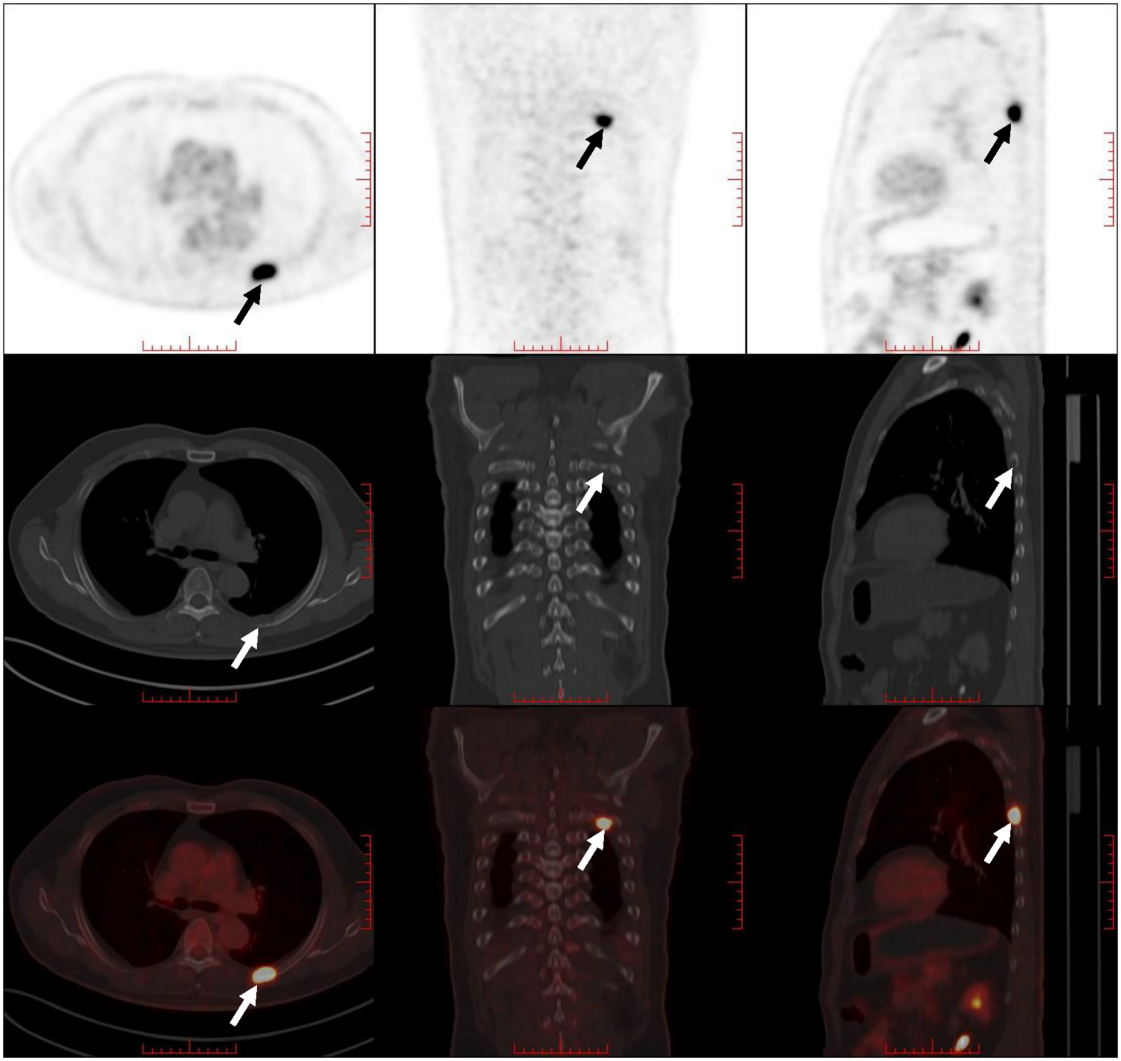
PET/CT image showed obvious osteolytic bone destruction and abnormal FDG uptake in the right fifth rib with local soft tissue mass formation (indicated by arrows).

**Figure 3:**
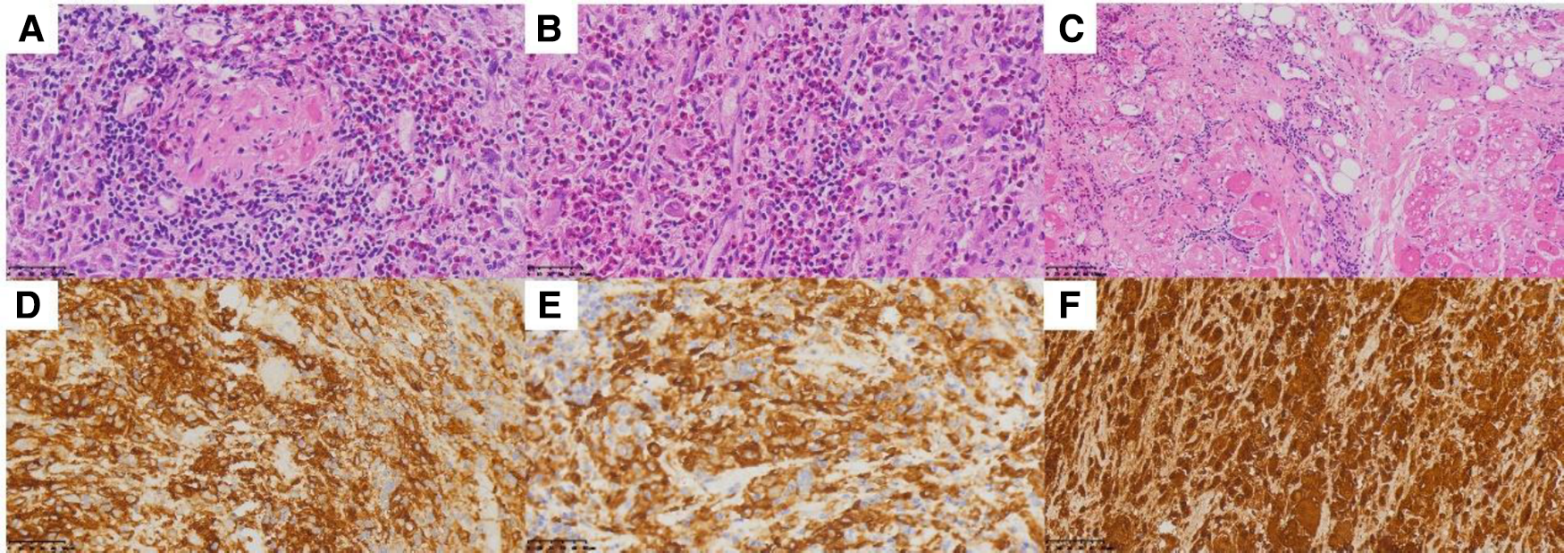
(**A–C**) Hematoxylin and eosin stain demonstrates histiocytes with pale cytoplasm and reniform nuclei. (**D**) CD1a, (**E**) Langerin, and (**F**) S-100.

**Figure 4:**
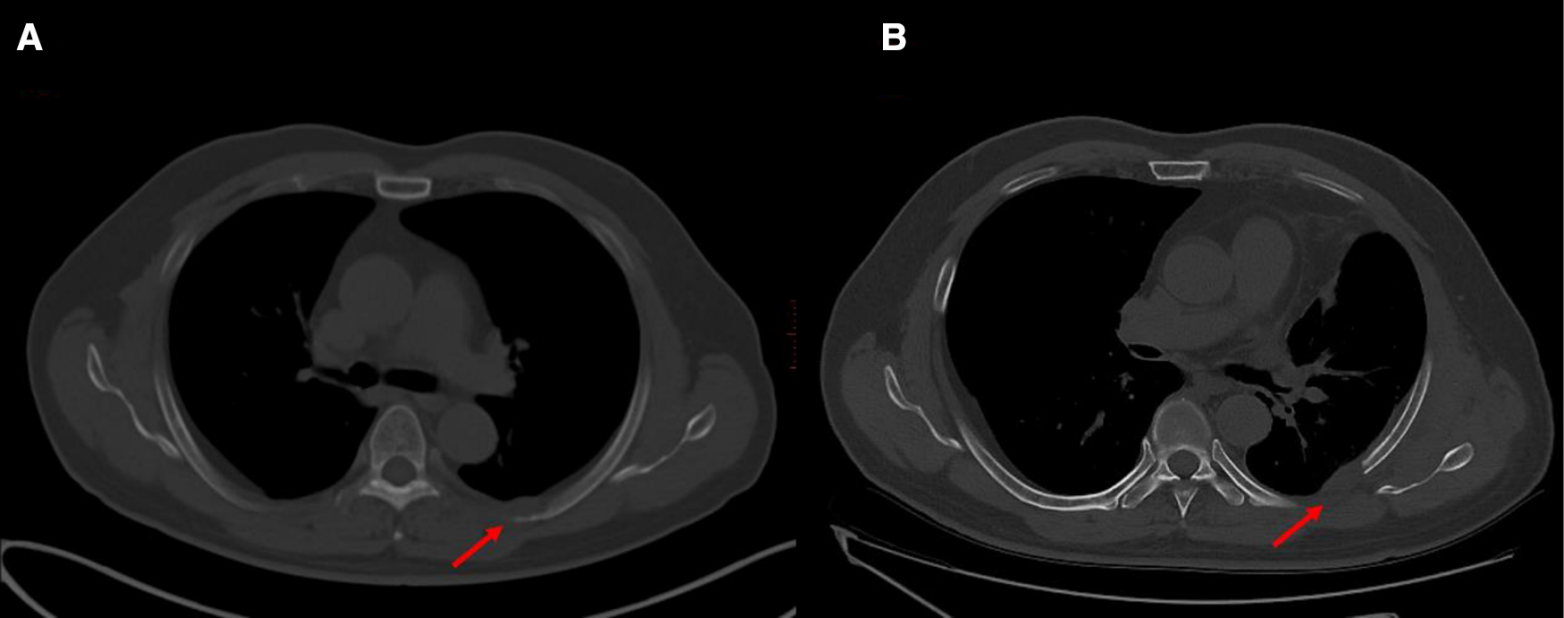
Images before and after surgery. (**A**) Prior to surgery, showing an osteolytic lesion in the left fifth rib. (**B**) One month after surgery, part of the fifth rib was excised with the lesion.

A 61-year-old male patient with a 15-day history of dull pain in the left chest was admitted to our hospital. He had a 6-year history of hypertension and was treated with valsartan and amlodipine tablets every day. Upon laboratory examination, the T-cell spot of tuberculosis assay (T-SPOT.TB) was positive and other laboratory indicators were normal. No obvious abnormalities were found in physical examination. Plain chest CT showed an osteolytic lesion in the left fifth rib. To evaluate the extent of skeletal lesions, ^99m^Tc-MDP SPECT was performed. A lesion with high uptake of ^99m^Tc-MDP in the left fifth rib was revealed and no other lesions were found in the image (Figure 1). These two imaging examinations suggested that the lesion might be malignant process. Therefore,^18^ F-FDG PET/CT was performed for staging. PET/CT image showed obvious osteolytic bone destruction and abnormal FDG uptake (SUVmax 14.5) in the left fifth rib with local soft tissue mass formation (Figure 2). With no other malignant signs found on PET/CT, the patient underwent a rib biopsy to confirm the diagnosis. Unexpectedly, histopathological examination suggested that the lesion was an eosinophilic granuloma. LCH was considered. Subsequently, the lesion was approached through a left posterolateral thoracotomy, and part of the fifth rib was excised with the lesion. By immunohistochemistry stain, the cells of surgical specimens were positive for CD1a, S-100, and Langerin, which confirmed the diagnosis of LCH (Figure 3). The patient is currently in good health condition without chest pain after the operation and does not present any complications during follow-up. The relevant images are shown in Figure 4.

## Discussion

LCH is a rare disease characterized by the proliferation and infiltration of Langerhans cells. It commonly occurs in children, and the incidence in adults is about one to two cases per million. In the case we presented, the patient was 61 years old, and it is a rare case for the age.

Classically, LCH was defined as three different kinds of syndromes: eosinophilic granuloma, Abt–Letterer–Siwe disease, and Hand–Schuller–Christian disease. Currently, LCH has been described as a broad spectrum of diseases ranging from local single-system lesions to disseminated multisystem diseases based on the number of involved sites, the site of lesions, and whether the disease is involving “risk organs” (e.g., hematopoietic system, liver, and/or spleen). The skull is the most commonly involved of the skeleton system, which occurs in more than two-thirds of patients, followed by the spine, limbs, and pelvis (10). The lesion of our patient was isolated, which was located in the rib; thus, it was considered an eosinophilic granuloma.

A biopsy of the involved site is suggested to confirm the diagnosis. The histological features of LCH are various, and the primary lesion is granulomatous with polymorphic infiltrate of mixed LCs, T lymphocytes, macrophages, multinucleated giant cells, and eosinophils (11). The cells of LCH represent positive immunohistochemistry for CD1a, Langerin (CD207), and S-100 protein. Nowadays, the detection of BRAFV600E in plasma and urine has been used to aid in the diagnosis of LCH, assessment of therapeutic efficacy, and detection of recurrent cases (12). In the present case, the patient underwent rib resection, and the cells of the lesion were positive for CD1a, Langerin, and S-100 by immunohistochemistry stain, which supported the diagnosis of LCH.

Imaging modalities, such as plain radiography, CT, MRI, PET/CT, and bone scintigraphy, are useful in the diagnosis and follow-up of patients with LCH. Plain radiography can reveal single or multiple aggressive-looking lytic lesions when the skeleton is involved, especially in the early stage (13). CT scanning is used in providing details of any cortical destruction and soft-tissue involvement. MR imaging is better suited for bone marrow, the extent of the soft-tissue lesions, and their relationship with adjacent structures. Bone scintigraphy is often proposed to evaluate the extent of skeletal lesions. ^18^F-FDG PET/CT has been proven to have a greater accuracy in identifying active lesions and is now being routinely used to monitor the disease in the initial evaluation and follow-up (14). A study conducted by Albano et al. found that the average SUVmax (maximum standardized uptake value) of bone lesions of LCH was 1.9–16.2 (15). In the case we presented, the imaging features were consistent with LCH. The SUVmax of the lesion was 14.8. However, other diseases should also be considered, such as osteomyelitis, osteochondroma, plasmacytoma, and Ewing's sarcoma.

The treatment of LCH remains controversial for its various clinical characteristics and the lack of standard diagnostic and evaluation criteria. Observation or surgical excision is recommended when the disease is localized. However, if high-risk organs (liver, spleen, bone marrow) are involved or in the case of single-system disease with multiple lesions or multisystem diseases, systemic chemotherapy is suggested (16). According to the clinical symptoms, imaging characteristics, and pathological findings of the lesion biopsy, our patient was considered to have isolated rib LCH, which was classified as a single-system disease without high-risk organ involvement. Thus, he underwent rib surgery with no chemotherapy administered.

## Conclusions

In conclusion, we present here a rare and unusual case of an adult with isolated rib LCH. LCH most frequently occurs in children while our patient is a 61-year-old male. Differential diagnosis should include osteomyelitis, osteochondroma, plasmacytoma, and Ewing's sarcoma before the results of histopathology especially immunohistochemical phenotype.^18^F-FDG PET/CT is useful for the diagnosis, staging, and prognostic evaluation of LCH, especially for identifying occult multiorgan involvement. ^18^F-FDG PET/CT has been routinely used to monitor diseases during treatment. Although single-site and single-system LCH of the rib are rare in adults, it can be treated well with surgery and systemic chemotherapy is not suggested. LCH should not be neglected in the diagnosis of an adult with a lytic rib lesion.

## Data Availability

The original contributions presented in the study are included in the article/Supplementary Material, further inquiries can be directed to the corresponding author.
